# Aggregating Knockoffs for False Discovery Rate Control with an Application to Gut Microbiome Data

**DOI:** 10.3390/e23020230

**Published:** 2021-02-16

**Authors:** Fang Xie, Johannes Lederer

**Affiliations:** Department of Mathematics, Ruhr-University Bochum, Universitätsstraße 150, 44801 Bochum, Germany; johannes.lederer@rub.de

**Keywords:** false discovery rate control, knockoffs, variable selection, gut microbiome

## Abstract

Recent discoveries suggest that our gut microbiome plays an important role in our health and wellbeing. However, the gut microbiome data are intricate; for example, the microbial diversity in the gut makes the data high-dimensional. While there are dedicated high-dimensional methods, such as the lasso estimator, they always come with the risk of false discoveries. Knockoffs are a recent approach to control the number of false discoveries. In this paper, we show that knockoffs can be aggregated to increase power while retaining sharp control over the false discoveries. We support our method both in theory and simulations, and we show that it can lead to new discoveries on microbiome data from the American Gut Project. In particular, our results indicate that several phyla that have been overlooked so far are associated with obesity.

## 1. Introduction

Research on associations between the microbiome in the human gut and health and disease has surged in recent years [1,2,3,4]. Data on the microbiome are abundant in view of citizen science endeavors such as the American Gut Project [5], but these non-standard ways of data collection limit data quality.

Another difficulty in the analysis of microbiome data is the high dimensionality, which means that the number of parameters is large. The high-dimensionality is due to the diversity of the microbiome: at the phylum level, there are typically several dozen types of microbes; at the genus level, there are even several hundred types of microbes. Finding the microbes that are connected to a trait, therefore, requires the use of variable selection techniques. Consequently, in view of the low data quality and the high dimensionality, research on microbiome data is in particular need for controlling false discovery rates.

As a specific example where false discovery control can be important, we are interested in finding phyla and genera of microbes that are related to obesity. Successful detection of such groups of microbes could eventually lead to new means for weight control. We model the task as a variable selection problem in logistic regression with the obesity as the dependent variable and the (log-transformed) counts of the microbial relative abundancies as the variables. The number of parameters, that is, the number of potential phyla/genera, is large, while the number of actually relevant phyla/genera is assumed to be small; hence, we speak of sparse, high-dimensional logistic regression. A number of corresponding variable selection methods have been established, including especially $\ell_{1}$-penalized logistic regression [6], which can be equipped with knockoffs to do FDR control [7]. However, in our application, these standard pipelines perform insufficiently: for example, phyla that are known to be associated with obesity are missed, and few phyla are selected in the first place.

To increase the variable selection performance, we propose a simple aggregation scheme. It consists of two steps: the knockoff method is run *k* times with a decreasing FDR target level, and the selections are then combined. We show that this aggregation scheme keeps FDR control intact while improving power in practice.

Our three key contributions are:

1. We introduce an aggregation scheme that provably retains the original methods’ guarantees—see Theorem 1.
2. We show numerically that the aggregation can increase the original methods’ power—see Section 3.1 and Section 3.2.
3. We show that the resulting pipelines for FDR control can be readily applied to empirical data and lead to new discoveries—see Section 3.3.

The remainder of the paper is organized as follows. In Section 2, we introduce our pipeline and establish its theory. In Section 3.1 and Section 3.2, we verify the accuracy of the pipeline in simulations. In Section 3.3, we then show the usefulness of the pipeline in selecting phyla and genera connected with obesity. In Section 4, we finally conclude with a brief discussion.

## 2. Methods and Theory

In this section, we introduce and study our aggregation scheme for knockoffs. We presume *n* independent data pairs $\left(x_{1},y_{1}\right),\cdots ,\left(x_{n},y_{n}\right)$, where each $x_{i}\in R^{p}$ is a vector of variables and $y_{i}\in R$ an outcome. We keep the relationship between the outcomes and variables completely general at this point—the relationship could be linear, logistic, or anything else—but we assume that this relationship is captured by a parameter $\beta \in R^{p}$. Our target for inference is then the active set $S^{*}:=\left\{j:\beta_{j}\neq 0\right\}$.

Two important quality measures for an estimate ${\hat{S}}_{q}\left[X,y\right]$ of $S^{*}$ are the FDR level

$$\mathrm{FDR}:=E\left[\frac{|{\hat{S}}_{q}\left[X,y\right]\backslash S^{*}|}{|{\hat{S}}_{q}\left[X,y\right]|\vee 1}\right]$$

and the power

$$\mathrm{power}:=E\left[\frac{|{\hat{S}}_{q}\left[X,y\right]\cap S^{*}|}{|{\hat{S}}_{q}\left[X,y\right]|\vee 1}\right]\;,$$

where |·| denotes the cardinality of a set. Our focus is on estimators that provide FDR control at level q∈[0,1], that is,

(1) FDR≤q,

while having large power. In the following, we recall that the knockoff filter provides FDR control. We then equip the knockoff filter with an aggregation step to improve its power.

### 2.1. A Brief Introduction to the Knockoff Filter

The main ingredient of the knockoff method [7] is a "knockoff version" $\tilde{X}\in R^{n\times p}$ of the design matrix X. This new matrix $\tilde{X}$ is essentially X with additional noise such that (1) the estimator can distinguish predictors from X and $\tilde{X}$ but (2) both design matrices still have a similar correlation structure. The idea is then to compare the selections of predictors in $\tilde{X}$ and in X when estimating on the extended design $\left[\tilde{X}\;X\right]$.

Denoting $\tilde{X}={\left({\tilde{x}}_{1},\ldots ,{\tilde{x}}_{n}\right)}^{⊤}$ and $X={\left(x_{1},\ldots ,x_{n}\right)}^{⊤}$, the underlying assumption is that $x_{i}∼N\left(0_{p},\Sigma \right)$ for a positive definite matrix $\Sigma \in R^{p\times p}$. The knockoffs are then generated according to [8]

(2) $${\tilde{x}}_{i}|x_{i}∼N\left(\mu_{i},V\right)\;\;\mathrm{for}\;\mathrm{each}\;i\in \left\{1,\ldots ,n\right\}$$

with

$$\begin{array}{cc} \mu_{i} & :=x_{i}-x_{i}\Sigma^{-1}\mathrm{diag}\left\{a\right\}\;,\\ V & :=2\mathrm{diag}\left\{a\right\}-\mathrm{diag}\left\{a\right\}\Sigma^{-1}\mathrm{diag}\left\{a\right\}\;, \end{array}$$

where $a\in R^{p}$ is such that V is positive definite.

The variable selection is then based on the estimator

(3) $$\hat{\beta }\left[\tau ,X,\tilde{X},y\right]\in \arg\min_{\alpha \in R^{2p}}\left\{\;l\left[\alpha |[X\;\tilde{X}],y\right]+\sum_{j=1}^{2p}h_{\tau }\left[\alpha_{j}\right]\;\right\}\;,$$

where $l:R^{p}\mapsto R$ is a loss function, $\left[X\;\tilde{X}\right]\in R^{n\times 2p}$ the extended design matrix, and $h_{\tau }:R\mapsto \left[0,\infty \right)$ a penalty function with tuning parameter τ>0. Examples include the lasso (where $l\left[\alpha |[X\;\tilde{X}],y\right]:=\left|\;\right|y-[X\;\tilde{X}]\alpha {\left|\;\right|}_{2}^{2}$ and $h_{\tau }\left[\alpha_{j}\right]:=\tau \left|\alpha_{j}\right|$) and the penalized logistic regression (where $l\left[\alpha |[X\;\tilde{X}],y\right]=-\sum_{i=1}^{n}\left(y_{i}x_{i}^{⊤}\alpha -\ln\left(1+\exp\left(x_{i}^{⊤}\alpha \right)\right)\right)$ and $h_{\tau }\left[\alpha_{j}\right]:=\tau \left|\alpha_{j}\right|$).

The basic test statistics of the knockoff approach then capture the maximal tuning parameter of each variable entering the model:

$$\begin{array}{cc} Z_{j}\left[X,y\right] & :=\sup\left\{\;\tau :{\hat{\beta }}_{j}\left[\tau ,X,\tilde{X},y\right]\neq 0\;\right\}\\ {\tilde{Z}}_{j}\left[X,y\right] & :=\sup\left\{\;\tau :{\hat{\beta }}_{p+j}\left[\tau ,X,\tilde{X},y\right]\neq 0\;\right\} \end{array}$$

for j∈{1,…,p}. We surpress the dependence on [X,y] in the following for notational ease. The final test statistic $W:={\left(W_{1},\ldots ,W_{p}\right)}^{⊤}$ then combines the basic test statistics into

$$W_{j}:=\max\left\{Z_{j},{\tilde{Z}}_{j}\right\}\cdot \mathrm{sign}\left(Z_{j}-{\tilde{Z}}_{j}\right)\;\;\;\mathrm{for}\;j\in \left\{1,\ldots ,p\right\}\;.$$

This statistic compares how much earlier the original predictor enters the model as compared to the fake predictor—or the other way around. The threshold of the standard knockoff procedure for a given FDR level q∈[0,1] is then

$$T_{q}:=\min\left\{\;t\in W:\frac{\#\{j:W_{j}\leq -t\}}{\#\{j:W_{j}\geq t\}\vee 1}\leq q\;\right\}\;,$$

where $W:=\{|W_{j}|:j\in \left\{1,\ldots ,p\right\}\}$.

The knockoff procedure has another variant, called knockoff+, which differs in the threshold:

$$T_{q}^{+}:=\min\left\{\;t\in W:\frac{1+\#\{j:W_{j}\leq -t\}}{\#\{j:W_{j}\geq t\}\vee 1}\leq q\;\right\}\;.$$

These definitions finally yield the active sets

$$\begin{array}{cc} {\hat{S}}_{q} & :=\left\{\;j:W_{j}\geq T_{q}\;\right\}\;,\\ {\hat{S}}_{q}^{+} & :=\left\{\;j:W_{j}\geq T_{q}^{+}\;\right\}\;. \end{array}$$

The active sets ${\hat{S}}_{q}^{+}$ indeed provide FDR control at level *q*, that is, satisfy inequality (1)—see [7], [Theorem 2]; the active sets ${\hat{S}}_{q}$ provide an approximate version of it—see [7], [Theorem 1].

### 2.2. Aggregating Knockoffs

We now introduce the aggregation scheme and its theory. The aggregation scheme applies the knockoff method *k* times and combines the results:

*Step 1: Given a target FDR q∈[0,1], choose a sequence $q_{1},\ldots ,q_{k}\in \left[0,1\right]$ such that $q=\sum_{i=1}^{k}q_{i}$. Apply the standard knockoff (or knockoff+) procedure k times, once for each target FDR $q_{i}$, and denote the corresponding k-estimated active sets by ${\hat{S}}_{q_{1}},\ldots ,{\hat{S}}_{q_{k}}$ (or ${\hat{S}}_{q_{1}}^{+},\ldots ,{\hat{S}}_{q_{k}}^{+}$).*

*Step 2: Combine these k-estimated active sets by taking the union:*

$${\hat{S}}_{q,\mathrm{AKO}}\left[k\right]:=\cup_{i=1}^{k}{\hat{S}}_{q_{i}}\;\;\;\;\left(\mathrm{or}\;{\hat{S}}_{q,\mathrm{AKO}}^{+}\left[k\right]:=\cup_{i=1}^{k}{\hat{S}}_{q_{i}}^{+}\right)\;.$$

The intuition behind this scheme is that increasing the number of knockoffs stabilizes the outcome and improves the power. While applied here to the knockoff method, we emphasize that the aggregation scheme can be applied to every model and estimator as long as the there is a method for FDR control to start with. Hence, rather than the standard knockoffs as used below, we could equally well use model-X knockoffs [9], deep knockoffs [10], or KnockoffGAN [11].

On the other hand, the aggregation scheme retains the knockoffs’ theoretical guarantees:

**Theorem** **1.** *Given a target FDR level q∈[0,1], the set ${\hat{S}}_{q,\mathrm{AKO}}^{+}\left[k\right]$ of the aggregation scheme for knockoff+ provides FDR control at level q:*

$$E\left[\frac{|{\hat{S}}_{q,\mathrm{AKO}}^{+}\left[k\right]\backslash S^{*}|}{|{\hat{S}}_{q,\mathrm{AKO}}^{+}\left[k\right]|\vee 1}\right]\leq q\;.$$

Similarly, the scheme retains the approximate FDR control of standard knockoffs—we will demonstrate this in our simulations.

**Proof** **of Theorem 1.** The proof is—maybe suprisingly—simple. By Theorem 2 in [7], we have for all $q_{i}$, i∈{1,…,k},

$$E\left[\frac{|{\hat{S}}_{q_{i}}^{+}\backslash S^{*}|}{|{\hat{S}}_{q_{i}}^{+}|\vee 1}\right]\leq q_{i}\;.$$

Hence,

$$\begin{array}{cc} E\left[\frac{|{\hat{S}}_{q,\mathrm{AKO}}^{+}\left[k\right]\backslash S^{*}|}{|{\hat{S}}_{q,\mathrm{AKO}}^{+}\left[k\right]|\vee 1}\right] & =E\left[\frac{|\left(\cup_{i=1}^{k}{\hat{S}}_{q_{i}}^{+}\right)\backslash S^{*}|}{|\cup_{i=1}^{k}{\hat{S}}_{q_{i}}^{+}|\vee 1}\right]\\ \; & =E\left[\frac{|\cup_{i=1}^{k}\left({\hat{S}}_{q_{i}}^{+}\backslash S^{*}\right)|}{|\cup_{i=1}^{k}{\hat{S}}_{q_{i}}^{+}|\vee 1}\right]\\ \; & \leq E\left[\sum_{i=1}^{k}\frac{|{\hat{S}}_{q_{i}}^{+}\backslash S^{*}|}{|\cup_{i=1}^{k}{\hat{S}}_{q_{i}}^{+}|\vee 1}\right]\\ \; & \leq E\left[\sum_{i=1}^{k}\frac{|{\hat{S}}_{q_{i}}^{+}\backslash S^{*}|}{|{\hat{S}}_{q_{i}}^{+}|\vee 1}\right]\\ \; & =\sum_{i=1}^{k}E\left[\frac{|{\hat{S}}_{q_{i}}^{+}\backslash S^{*}|}{|{\hat{S}}_{q_{i}}^{+}|\vee 1}\right]\\ \; & \leq \sum_{i=1}^{k}q_{i}=q\;, \end{array}$$

as desired. □

For k=1, our method equals the standard knockoff (or knockoff+) procedure; in practice, we recommend k≈5 as a trade-off between computational effort and statistical effect. We also recommend $q_{i}=q/2^{i-1}$, which, strictly speaking, does not meet our assumptions on $q_{1},\ldots ,q_{k}$ exactly, but it works well empirically—see the simulations.

### 2.3. Other Approaches

While preparing this manuscript, two other ways of aggregating knockoffs were proposed [12,13]. While we use *k* knockoffs in *k* processes individually and aggregate at the end, they use multiple knockoffs simultaneously in one process. They can also show that their schemes provide valid FDR control, but we can argue that our approach is considerably simpler. We illustrate in the Appendix A.1 that we typically get more power than the multiple knockoffs method proposed by [12]; we have not gotten the scheme of [13] to run yet.

## 3. Simulations and a Real Data Analysis

In this section, we test our method empirically both on synthetic and on real data. We incorporate the specifics of microbiome data: First, in line with recent proposals for generalized linear models in this context [14], we study linear regression as well as logistic regression, and we transform count data with the standard log-transformation [15]. Second, since gut microbiome data tends to be correlated [16], we ensure that the synthetic data are also correlated. Third, since gut microbime data also tend to be zero-inflated, we replace zero values by 0.5 times the observed minimum abundance, which is standard approach in microbiome analysis [17,18]. We compare our modified aggregating knockoff pipeline applied to $\ell_{1}$-penalized regression (called AKO henceforth) with the standard knockoff pipeline (KO).

Throughout, we set k=5 and $q_{i}=q/2^{i-1}$. We also show the results for other choice of $q_{1},\ldots ,q_{k}$ in the Appendix A.2.

### 3.1. Simulation 1: Linear Regression

We first consider linear regression. The dimensions of the synthetic data are (n,p)∈{(200,100),(400,200)}. The rows $x_{i}$ of the design matrix are sampled i.i.d. from $N(0_{p},\Sigma )$ with the elements in Σ satisfying $\Sigma_{ij}=\rho^{\left|i-j\right|}$ with correlation factor ρ=0.5. The noise is drawn from $u∼N(0,\sigma^{2}I_{n})$. The true parameter β has 20 nonzero coefficients, which are selected uniformly at random from {1,…,p} and set to 1 before the entire vector is rescaled to have signal-to-noise ratio ${\left|\;|X\beta |\;\right|}_{2}^{2}/n\sigma^{2}=5$ with $\sigma^{2}=1$. The outcome y is then generated by the linear model

y=Xβ+u.

The test statistic *W* is based on the lasso method as described in the preceeding section.

We compare the empirical FDR and power averaged over r=100 repetitions of the simulations for each method. The ideal result would be an average FDR of at most *q* and a power equal to 1.

**Figure 1:**
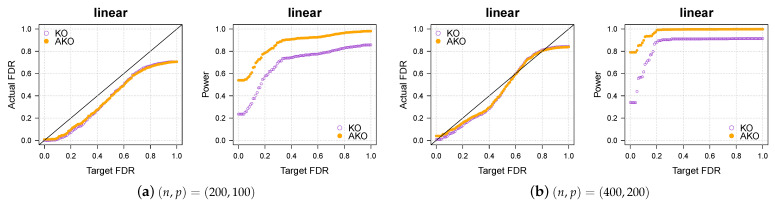
Our approach, AKO (solid, orange circles), has a similar FDR to the standard KO (hollow, purple circles) but has more power.

The results in Figure 1a,b show that our pipeline retains the KO’s FDR control while increasing the power.

### 3.2. Simulation 2: Logistic Regression

We now consider logistic regression (the paper [19] was the first one to apply our scheme to logistic regression). The above settings remain the same except for the outcomes $y_{i}$ being generated by

$$\mathrm{Pr}\left(y_{i}=1\right)=\frac{\exp(x_{i}^{⊤}\beta )}{1+\exp(x_{i}^{⊤}\beta )}\;\;\;\mathrm{for}\;i\in \left\{1,\ldots ,n\right\}.$$

**Figure 2:**
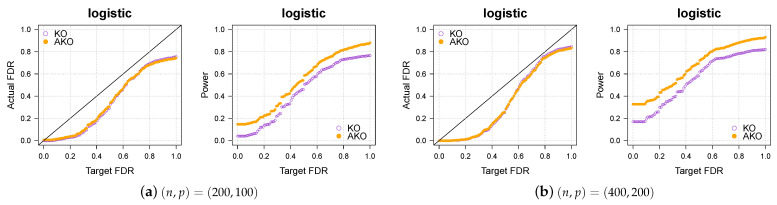
Our approach AKO (solid, orange circles) has a similar FDR to the standard KO (hollow, purple circles) but has more power.

The results in Figure 2a,b show again that our pipeline retains the KO’s FDR control while increasing the power. For more simulations in various settings, please refer to Appendix A.3.

### 3.3. Influence of the Gut Microbiome on Obesity

A well-functioning gut microbiome is essential for health [2]; for example, there is strong evidence that the composition of the microbiome is connected to obesity [20,21,22]. Existing research about this connection has focused only on phyla of bacteria that are abundant in most guts, such as *Actinobacteria*, *Bacteroidetes*, *Firmicutes*, and *Verrucomicrobia* [3,23,24,25,26]. In the following analysis, in contrast, we include the microbiome in its entirety. Our findings suggest that also phyla beyond those ones mentioned in the literature are connected to obesity.

The data for our analysis are from the American Gut Project [5]. The scope is American adults with age between 20 and 69 and BMI between $15\;\mathrm{kg}/m^{2}$ and $60\;\mathrm{kg}/m^{2}$ in the collection up to January, 2018. This includes n=8404 subjects, of which 278 are underweight (uw; BMI below $18.5\;\mathrm{kg}/m^{2}$), 4972 have normal weight (nor; 18.5–25$\;\mathrm{kg}/m^{2}$), 2253 are overweight (ow; 25–30$\;\mathrm{kg}/m^{2}$), and 901 are obese (ob; above $30\;\mathrm{kg}/m^{2}$). We transform the data and deal with the zero-counts as decribed earlier. The total number of phyla in our scope is p=55.

The underlying model for these data is $\ell_{1}$-penalized logistic regression model with outcomes $y_{i}=1$ (ob) when BMI ≥30 and $y_{i}=0$ (non-ob) otherwise. The target FDR level is q=0.1. Seven different groupings are considered to get the most out of the data: (i) all four groups (uw+nor+ow+ob), (ii) uw+ob, (iii) nor+ob, (iv) ow+ob, (v) uw+nor+ob, (vi) uw+ow+ob and (vii) nor+ow+ob. The AKO is applied with $\left\{q_{i}=q/2^{i-1}:i\in \left\{1,\ldots ,k\right\}\right\}$.

The results in Table 1 show that our pipeline selects more phyla in general. Since the theory and the above similations suggest that both methods have similar FDR, the results indicate that our pipeline has more power. In particilar, phyla that are known to be connected with obesity, such as *Actinobacteria*, *Bacteroidetes*, *Firmicutes*, and *Verrucomicrobia* [3,23,24,25,26], are selected by AKO more often across the seven groupings.

The results also highlight *Spirochaetes*, which have not been associated with obesity in the literature, yet. The standard pipeline does not seem to have the power to select it, and similarly, two other known FDR control methods for microbiome data—the standard BH procedure [27] and the TreeFDR [28]—select *Spirochaetes* only for large FDR levels (q≳0.8). (Futher comparisons with the BH procedure, TreeFDR, and the plain KO can be found in the Appendix A.4.) In contrast, our method selects *Spirochaetes* even at very small FDR levels (such as q=0.01), which strongly suggests a connection between *Spirochaetes* and obesity.

Our pipeline can, of course, also be applied to more detailed taxonomic ranks. As an illustration, we report the results of an application to genera for ALL—cf. (i) in Table 1—in Table 2. The data sampling is same with the phyla analysis. The only difference is that the total number of genera is p=969. The rest of results for other six different groupings are given in the Appendix B. We find correspondingly that AKO selects more genera than the standard KO.

## 4. Discussion

Our aggregation scheme for knockoffs is supported by theory (Section 2) and simulations (Section 3.1 and Section 3.2) and may lead to new discoveries in microbiomics (Section 3.3).

While we focus on a specific pipeline, our concept applies very generally. For example, it does not depend on the underlying statistical model or estimator but only on the availability of FDR control. In particular, the FDR control can be established via standard knockoffs or any other scheme. This flexibility is particularly interesting in practice: while the standard knockoffs rely on normality, other knockoff procedures, such as model-X knockoffs [9], deep knockoffs [10], and KnockoffGAN [11], allow for much more general designs. Hence, the standard knockoffs can be readily swapped for those procedures in our pipeline (without any changes to the methodology or theory) when indicated in an application.

Considerably later than our paper has been put online, two other papers on the topic have also been put online [29,30]—apparently without being aware of our manuscript. A comparison to those results would also be of interest.

## Figures and Tables

**Table 1:** Selected bacterial phyla by our pipeline (AKO) and the original pipeline (KO) at FDR level q=0.1 for seven groupings. AKO consistently selects more phyla than KO.

**(i) all**		**(ii) uw + ob**	
**KO**	**AKO**	**KO**	**AKO**
*Actinobacteria*	*Actinobacteria*	*Actinobacteria*	*Actinobacteria*
	*Bacteroidetes*		
*Cyanobacteria*	*Cyanobacteria*		*Cyanobacteria*
			*Firmicutes*
*Proteobacteria*	*Proteobacteria*		
	*Spirochaetes*		
*Synergistetes*	*Synergistetes*		*Synergistetes*
*Tenericutes*	*Tenericutes*	*Tenericutes*	*Tenericutes*
	*Verrucomicrobia*		
**(iii) nor + ob**		**(iv) ow + ob**	
**KO**	**AKO**	**KO**	**AKO**
*Actinobacteria*	*Actinobacteria*		*Actinobacteria*
*Bacteroidetes*	*Bacteroidetes*		
*Cyanobacteria*	*Cyanobacteria*	*Cyanobacteria*	*Cyanobacteria*
			*Firmicutes*
	*Lentisphaerae*		
*Proteobacteria*	*Proteobacteria*		*Proteobacteria*
	*Spirochaetes*		*Spirochaetes*
*Synergistetes*	*Synergistetes*		*Synergistetes*
			*TM7*
*Tenericutes*	*Tenericutes*	*Tenericutes*	*Tenericutes*
	*Verrucomicrobia*		
	*Thermi*		
**(v) uw + nor + ob**		**(vi) uw + ow + ob**	
**KO**	**AKO**	**KO**	**AKO**
*Actinobacteria*	*Actinobacteria*		*Actinobacteria*
*Bacteroidetes*	*Bacteroidetes*		
*Cyanobacteria*	*Cyanobacteria*	*Cyanobacteria*	*Cyanobacteria*
			*Firmicutes*
	*Lentisphaerae*		
*Proteobacteria*	*Proteobacteria*		*Proteobacteria*
	*Spirochaetes*		*Spirochaetes*
*Synergistetes*	*Synergistetes*		*Synergistetes*
			*TM7*
*Tenericutes*	*Tenericutes*	*Tenericutes*	*Tenericutes*
**(vii) nor+ow+ob**			
**KO**	**AKO**		
	*Actinobacteria*		
	*Bacteroidetes*		
*Cyanobacteria*	*Cyanobacteria*		
*Proteobacteria*	*Proteobacteria*		
	*Spirochaetes*		
*Synergistetes*	*Synergistetes*		
*Tenericutes*	*Tenericutes*		
	*Verrucomicrobia*		

**Table 2:** Selected bacterial genera by our pipeline (AKO) and the original pipeline (KO) at FDR level q=0.1 for ALL—cf. (i) in Table 1. AKO selects more genera than the original KO.

Phylum	KO	AKO
*Actinobacteria*	*Actinomyces*	*Actinomyces*
	*Collinsella*	*Collinsella*
	*Eggerthella*	*Eggerthella*
*Cyanobacteria*	*YS2*	*YS2*
		*Streptophyta*
*Firmicutes*	*Bacillus*	*Bacillus*
		*Lactobacillus*
	*Lactococcus*	*Lactococcus*
		*Clostridium*
	*Lachnospira*	*Lachnospira*
	*Ruminococcus*	*Ruminococcus*
		*Peptostreptococcaceae*
	*Acidaminococcus*	*Acidaminococcus*
	*Megasphaera*	*Megasphaera*
		*Mogibacteriaceae*
	*Erysipelotrichaceae*	*Erysipelotrichaceae*
	*Catenibacterium*	*Catenibacterium*
*Proteobacteria*	*RF32*	*RF32*
	*Haemophilus*	*Haemophilus*
*Tenericutes*		*RF39*

**Figure A1:**
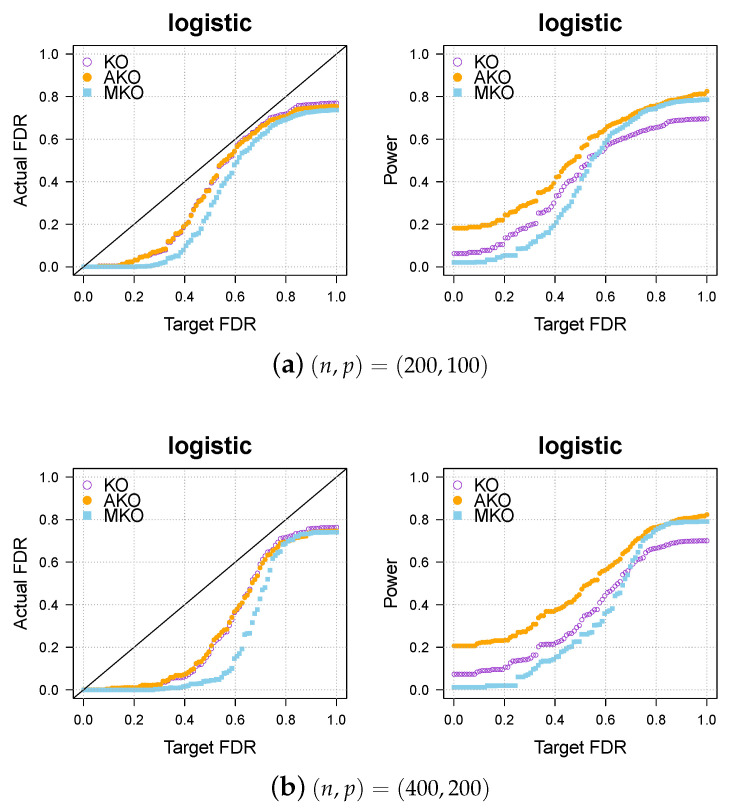
Our approach AKO (solid, orange circles) has a similar FDR to the standard KO (hollow, purple circles) but has more power. The MKO (solid, blue square) is more conservative than our AKO, has lower power.

**Figure A2:**
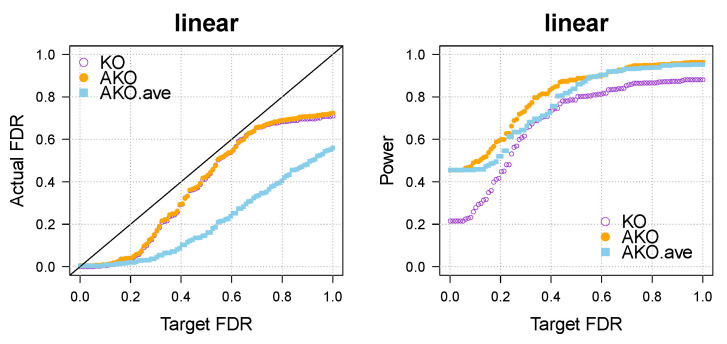
Actual FDR and power of the KO, AKO, AKO.ave in the linear case with (n,p)=(200,100).

**Figure A3:**
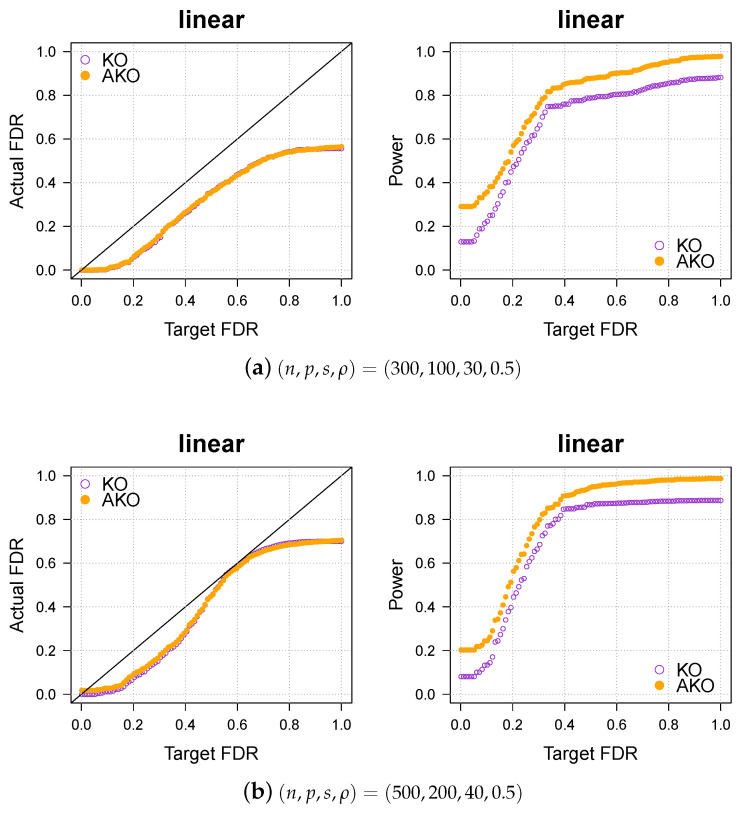
Our approach AKO (solid, orange circles) has a similar FDR to the standard KO (hollow, purple circles) but has more power.

## Data Availability

All the software, simulations, and data analyses are provided at github.com/lederlab (accessed on 20 January 2021). The data for our analysis are downloaded from the website of the American Gut Project (accessed on 20 January 2021), which is public.

## Appendix A. Additional Explanations

### Appendix A.1. Further Simulations for Comparison to Multiple Knockoffs (MKO)

We compare the three methods—KO, AKO, and MKO—in the logistic cases. The results shown in Figure A1 indicate that the MKO is more conservative than both our pipeline and the KO, and our pipeline always has higher power than KO and MKO.

### Appendix A.2. Choice of q_1_,…,q_k_

The theory applies to every choice of $q_{1},\cdots ,q_{k}$ that satisfies the condition in step 1 on page 3, but indeed, each choice has slightly different characteristics in practice. To give an example, we have added the result for $q_{i}=q/k$ (AKO.ave, Figure A2) to Figure 1a in the main text. We observe in general that the results do look different for each choice of the sequence but that the differences are usually only small.

### Appendix A.3. Various Settings for the Simulation Part

Our conclusions hold over a wide spectrum of settings, including different dimensionalities, sparsity levels, correlations, and so forth. To illustrate this, we vary the settings of the linear case (see Figure 1a,b) once more in Figure A3 below.

### Appendix A.4. Better Than other Competitors (under the AGP Data)

Knockoff methods seem to be better suited for the microbiome data than their competitors. For illustration, we have included BH [27] and TreeFDR [28] in the application that corresponds to Table 1 (i) in the paper. We find (in Table A1) on those data more generally that the knockoff methods select many more predictors than other methods.

**Table A1:** Selected bacterial phyla by four methods—BH, TreeFDR, KO, and AKO (correponds to Table 1 (i)).

(i) ALL			
BH	TreeFDR	KO	AKO
		*Actinobacteria*	*Actinobacteria*
			*Bacteroidetes*
	*Cyanobacteria*	*Cyanobacteria*	*Cyanobacteria*
		*Proteobacteria*	*Proteobacteria*
			*Spirochaetes*
		*Synergistetes*	*Synergistetes*
		*Tenericutes*	*Tenericutes*
*Verrucomicrobia*	*Verrucomicrobia*		*Verrucomicrobia*

## Appendix B. Additional Results on the Genera Rank

We present in this section the results on the genera rank of different groupings; see Table A2, Table A3, Table A4, Table A5, Table A6 and Table A7.

**Table A2:** Analysis at the genus level rank for the grouping (ii) uw+ob. AKO selects more genera than the original KO.

Phylum	KO	AKO
Actinobacteria	Collinsella	Collinsella
Firmicutes		Lachnospira
		Acidaminococcus
		Catenibacterium
Tenericutes	RF39	RF39

**Table A3:** Analysis at the genus level for the grouping (iii) nor + ob. AKO selects more genera than the original KO.

Phylum	KO	AKO
Actinobacteria	Actinomyces	Actinomyces
	Collinsella	Collinsella
Cyanobacteria	YS2	YS2
Firmicutes	Bacillus	Bacillus
		Lactococcus
	Lachnospira	Lachnospira
	Ruminococcus	Ruminococcus
	Acidaminococcus	Acidaminococcus
	Megasphaera	Megasphaera
		Mogibacteriaceae
		Erysipelotrichaceae
	Catenibacterium	Catenibacterium
Proteobacteria	RF32	RF32
		Haemophilus
Tenericutes	RF39	RF39
		ML615J-28

**Table A4:** Analysis at the genus level for the grouping (iv) ow + ob. AKO selects more genera than the original KO.

Phylum	KO	AKO
Actinobacteria	Eggerthella	Eggerthella
Cyanobacteria	YS2	YS2
	Streptophyta	Streptophyta
Firmicutes		Bacillus
	Clostridium	Clostridium
	Lachnospira	Lachnospira
	Acidaminococcus	Acidaminococcus
		1-68
	Erysipelotrichaceae	Erysipelotrichaceae
		Catenibacterium
Proteobacteria	Haemophilus	Haemophilus

**Table A5:** Analysis at the genus level for the grouping (v) uw + nor + ob. AKO selects more genera than the original KO.

Phylum	KO	AKO
Actinobacteria	Actinomyces	Actinomyces
	Collinsella	Collinsella
Cyanobacteria	YS2	YS2
Firmicutes	Bacillus	Bacillus
		Lactococcus
	Lachnospira	Lachnospira
	Ruminococcus	Ruminococcus
	Acidaminococcus	Acidaminococcus
	Megasphaera	Megasphaera
		Mogibacteriaceae
		SHA-98
		Erysipelotrichaceae
	Catenibacterium	Catenibacterium
Proteobacteria	RF32	RF32
		Haemophilus
Tenericutes	RF39	RF39
		ML615J-28

**Table A6:** Analysis at the genus level for the grouping (vi) uw + ow + ob. AKO selects more genera than the original KO.

Phylum	KO	AKO
Actinobacteria	Eggerthella	Eggerthella
Cyanobacteria	YS2	YS2
	Streptophyta	Streptophyta
Firmicutes	Bacillus	Bacillus
		Lactobacillus
	Clostridium	Clostridium
	Lachnospira	Lachnospira
		Veillonellaceaes
	Acidaminococcus	Acidaminococcus
	1-68	1-68
	Erysipelotrichaceae	Erysipelotrichaceae
	Catenibacterium	Catenibacterium
	Eubacterium	Eubacterium
Proteobacteria		RF32
	Haemophilus	Haemophilus

**Table A7:** Analysis at the genus level for the grouping (vii) nor+ow+ob. AKO selects more genera than the original KO.

Phylum	KO	AKO
Actinobacteria	Actinomyces	Actinomyces
	Collinsella	Collinsella
	Eggerthella	Eggerthella
Cyanobacteria	YS2	YS2
Firmicutes	Bacillus	Bacillus
	Lachnospira	Lachnospira
	Ruminococcus	Ruminococcus
	Acidaminococcus	Acidaminococcus
	Megasphaera	Megasphaera
	Erysipelotrichaceae	Erysipelotrichaceae
	Catenibacterium	Catenibacterium
Proteobacteria	RF32	RF32
	Haemophilus	Haemophilus
Tenericutes		RF39

## References

[1] Evans J.M., Morris L.S., Marchesi J.R. (2013). The gut microbiome: The role of a virtual organ in the endocrinology of the host. J. Endocrinol..

[2] Huttenhower C., Gevers D., Knight R., Abubucker S., Badger J.H., Chinwalla A.T., Creasy H.H., Earl A.M., FitzGerald M.G., Fulton R.S. (2012). The Human Microbiome Project Consortium: Structure, function and diversity of the healthy human microbiome. Nature.

[3] Koliada A., Syzenko G., Moseiko V., Budovska L., Puchkov K., Perederiy V., Gavalko Y., Dorofeyev A., Romanenko M., Tkach S. (2017). Association between body mass index and Firmicutes/Bacteroidetes ratio in an adult Ukrainian population. BMC Microbiol..

[4] Ley R.E., Turnbaugh P.J., Klein S., Gordon J.I. (2006). Microbial ecology: Human gut microbes associated with obesity. Nature.

[5] Knight Lab American Gut Project. http://americangut.org.

[6] Ng A.Y. Feature selection, L 1 vs. L 2 regularization, and rotational invariance. Proceedings of the 21st International Conference on Machine Learning.

[7] Barber R.F., Candès E.J. (2015). Controlling the false discovery rate via knockoffs. Ann. Stat..

[8] Barber R.F., Candès E.J., Samworth R.J. (2018). Robust inference with knockoffs. arXiv.

[9] Candès E.J., Fan Y., Janson L., Lv J. (2018). Panning for gold: ‘Model-X’knockoffs for high dimensional controlled variable selection. J. R. Stat. Soc. Ser. (Stat. Methodol.).

[10] Romano Y., Sesia M., Candès E.J. (2019). Deep Knockoffs. J. Am. Stat. Assoc..

[11] Jordon J., Yoon J., van der Schaar M. KnockoffGAN: Generating Knockoffs for Feature Selection using Generative Adversarial Networks. Proceedings of the International Conference on Learning Representations.

[12] Holden L., Hellton K.H. (2018). Multiple Model-Free Knockoffs. arXiv.

[13] Gimenez J.R., Zou J. Improving the stability of the knockoff procedure: Multiple simultaneous knockoffs and entropy maximization. Proceedings of the 22nd International Conference on Artificial Intelligence and Statistics.

[14] Lu J., Shi P., Li H. (2019). Generalized linear models with linear constraints for microbiome compositional data. Biometrics.

[15] Aitchison J. (1982). The statistical analysis of compositional data. J. R. Stat. Soc. Ser. (Methodol.).

[16] Naqvi A., Rangwala H., Keshavarzian A., Gillevet P. (2010). Network-based modeling of the human gut microbiome. Chem. Biodivers..

[17] Aitchison J. (2003). The Statistical Analysis of Compositional Data.

[18] Kurtz Z.D., Müller C.L., Miraldi E.R., Littman D.R., Blaser M.J., Bonneau R.A. (2015). Sparse and Compositionally Robust Inference of Microbial Ecological Networks. PLoS Comput. Biol..

[19] Klose S., Lederer J. (2020). A Pipeline for Variable Selection and False Discovery Rate Control With an Application in Labor Economics. arXiv.

[20] Escobar J.S., Klotz B., Valdes B.E., Agudelo G.M. (2014). The gut microbiota of Colombians differs from that of Americans, Europeans and Asians. BMC Microbiol..

[21] Gérard P. (2016). Gut microbiota and obesity. Cell. Mol. Life Sci..

[22] Turnbaugh P.J., Gordon J.I. (2009). The core gut microbiome, energy balance and obesity. J. Physiol..

[23] Bai J., Hu Y., Bruner D.W. (2019). Composition of gut microbiota and its association with body mass index and lifestyle factors in a cohort of 7-18 years old children from the American Gut Project. Pediatr. Obes..

[24] Clarke S.F., Murphy E.F., Nilaweera K., Ross P.R., Shanahan F., O’Toole P.W., Cotter P.D. (2012). The gut microbiota and its relationship to diet and obesity. Gut Microbes.

[25] Depommier C., Everard A., Druart C., Plovier H., Van Hul M., Vieira-Silva S., Falony G., Raes J., Maiter D., Delzenne N.M. (2019). Supplementation with Akkermansia muciniphila in overweight and obese human volunteers: A proof-of-concept exploratory study. Nat. Med..

[26] Gao X., Zhang M., Xue J., Huang J., Zhuang R., Zhou X., Zhang H., Fu Q., Hao Y. (2018). Body Mass Index Differences in the Gut Microbiota Are Gender Specific. Front. Microbiol..

[27] Benjamini Y., Hochberg Y. (1995). Controlling the false discovery rate: A practical and powerful approach to multiple testing. J. R. Stat. Soc. Ser. B.

[28] Xiao J., Cao H., Chen J. (2017). False discovery rate control incorporating phylogenetic tree increases detection power in microbiome-wide multiple testing. Bioinformatics.

[29] Srinivasan A., Xue L., Zhan X. (2020). Compositional knockoff filter for high-dimensional regression analysis of microbiome data. Biometrics.

[30] Nguyen T.B., Chevalier J.A., Thirion B., Arlot S. Aggregation of multiple knockoffs. Proceedings of the 37th International Conference on Machine Learning.

